# ALDH1A3 upregulation and spontaneous metastasis formation is associated with acquired chemoresistance in colorectal cancer cells

**DOI:** 10.1186/s12885-018-4758-y

**Published:** 2018-08-24

**Authors:** Erika Durinikova, Zuzana Kozovska, Martina Poturnajova, Jana Plava, Zuzana Cierna, Andrea Babelova, Roman Bohovic, Silvia Schmidtova, Miroslav Tomas, Lucia Kucerova, Miroslava Matuskova

**Affiliations:** 1 Cancer Research Institute, Biomedical Research Center of Slovak Academy of Sciences, Dubravska cesta 9, 845 05 Bratislava, Slovakia; 2 Institute of Pathological Anatomy, Faculty of Medicine, Comenius University, Sasinkova 4, 813 72 Bratislava, Slovakia; 3 Department of Surgical Oncology of Slovak Medical University, National Cancer Institute, Klenova 1, 831 01 Bratislava, Slovakia

**Keywords:** 5-fluorouracil, Chemoresistance, Colorectal cancer, Aldehyde dehydrogenase, Metastasis

## Abstract

**Background:**

Efficiency of colorectal carcinoma treatment by chemotherapy is diminished as the resistance develops over time in patients. The same holds true for 5-fluorouracil, the drug used in first line chemotherapy of colorectal carcinoma.

**Methods:**

Chemoresistant derivative of HT-29 cells was prepared by long-term culturing in increasing concentration of 5-fluorouracil. Cells were characterized by viability assays, flow cytometry, gene expression arrays and kinetic imaging. Immunomagnetic separation was used for isolation of subpopulations positive for cancer stem cells-related surface markers. Aldehyde dehydrogenase expression was attenuated by siRNA. In vivo studies were performed on SCID/bg mice.

**Results:**

The prepared chemoresistant cell line labeled as HT-29/EGFP/FUR is assigned with different morphology, decreased proliferation rate and 135-fold increased IC_50_ value for 5-fluorouracil in comparison to parental counterparts HT-29/EGFP. The capability of chemoresistant cells to form tumor xenografts, when injected subcutaneously into SCID/bg mice, was strongly compromised, however, they formed distant metastases in mouse lungs spontaneously. Derived cells preserved their resistance in vitro and in vivo even without the 5-fluorouracil selection pressure. More importantly, they were resistant to cisplatin, oxaliplatin and cyclophosphamide exhibiting high cross-resistance along with alterations in expression of cancer-stem cell markers such as CD133, CD166, CD24, CD26, CXCR4, CD271 and CD274. We also detected increased aldehyde dehydrogenase (ALDH) activity associated with overexpression of specific ALDH isoform 1A3. Its inhibition by siRNA approach partially sensitized cells to various agents, thus linking for the first time the ALDH1A3 and chemoresistance in colorectal cancer.

**Conclusion:**

Our study demonstrated that acquired chemoresistance goes along with metastatic and migratory phenotype and can be accompanied with increased activity of aldehyde dehydrogenase. We describe here the valuable model to study molecular link between resistance to chemotherapy and metastatic dissemination.

## Background

Colorectal cancer (CRC) belongs among the most common cause of cancer death in the world. Even though there has been a decline in incidence in the past two decades due to better cancer screening measures, the morbidity has not decreased as substantially as those with other types of cancer. This could be changed with expanding knowledge of the biology of colon cancer cells, particularly with the subset of chemoresistant cells [1]. Despite currently used therapeutic regimens that have significantly increased survival of patients with metastatic CRC, nearly all tumors become chemoresistant. The multidrug resistance can be caused by overexpression of ATP-binding cassette transporters (ABC transporters) - specific efflux pumps that decrease bioavailability of administered drug, as reviewed [2]. The resistance was proposed to be promoted also through soluble factors with progranulin identified as a potential mediator of chemoresistance [3], through activation of TGF-β pathway [4] or the mechanism of CXCR4/PI3K/Akt downnstream signaling [5].

Chemoresistant cells and cancer stem cells (CSC) may represent overlapping cell populations within tumor as CSC exhibit characteristics of cells resistant to standard chemotherapeutics, and chemoresistant cells also express markers of CSC (reviewed by [6, 7]). So far, there is no consensus as to the exact criteria defining CSC for CRC, as markers vary according to tumor type. Molecular markers tested for distinct solid tumors are reviewed by [8, 9], as well as their biologic characteristics with therapeutic significance [6] and involvement in metastatic process [10]. Isolation of CSC from colon carcinomas can be accomplished by selection of subpopulation based on expression of one or multiple cell surface markers, i.e. CD133, CD24, CD26, CD29, CD44, CD44v6, CD166, Lgr5 [11–13], and Wnt activity [14].

The activity of aldehyde dehydrogenase (ALDH) enzymes is widely used as a CSC marker in many types of cancer. ALDH1 has three main isoforms - ALDH1A1, ALDH1A2, and ALDH1A3, and is a marker of normal tissue stem cells and cancer stem cells, where it is involved in self-renewal, differentiation and self-protection as reviewed in [15]. It was proven [16] that high ALDH1 expression indicates a poor prognosis in CRC patients, and is correlated with T stage, N stage and tumor differentiation. Growing body of evidence indicates that ALDH1A3 has the potential to be used as a target for cancer diagnosis and therapy as reviewed by [17]. The correlation of ALDH1A3 with breast tumor [18, 19], gliomas [20, 21], neuroblastoma [22], and rat colon model of familial adenomatous polyposis [23] was shown.

The aim of presented study was to derive chemoresistant CRC cell line in vitro and analyze the association between chemoresistance and CSC phenotype. Moreover, we focused on detailed analysis of chemoresistant derivative of HT-29 cell line and here we show, that acquired chemoresistance concomitantly selected for the cells with capability to spontaneously metastasize. Moreover, we were able to show, that downregulation of ALDH1A3 partially sensitized the cells to chemotherapy.

## Methods

### Cell cultures

Human colon adenocarcinoma cell line HT-29 (ECACC no. 91072201; cell line was last tested and authenticated on August 2nd, 2017 by STR Profiling) was maintained in high glucose (4.5 g/l) Dulbecco’s modified Eagle medium (DMEM; PAN Biotech, Germany) supplemented with 10% fetal calf serum (FCS; Biochrom AG, Germany), 2 mM glutamine (PAA Laboratories GmbH, Austria) or GlutaMAX (Gibco by Life Technologies, USA), and addition of antibiotics 10 μg/ml gentamicin (Sandoz, Germany) and 2.5 μg/ml amphotericin B (Sigma-Aldrich, USA). Tumor cell line HT-29 was retrovirally transduced to stably express enhanced green fluorescent protein (EGFP) as described in [24], selected based on G418 resistance (Geneticin; Serva, Germany), and evaluated by flow cytometry. Cells stably resistant to 5-fluorouracil (5-FU; Sigma-Aldrich) were developed by exposing parental chemonaïve cell line HT-29/EGFP to gradually increasing concentrations of this chemotherapeutic according to [25]. Finally, the 5-FU concentration was increased to the clinically relevant plasma concentration of 2 μg/ml, and the surviving chemoresistant cells were referred to as HT-29/EGFP/FUR. These cells were maintained in high glucose DMEM supplemented with 10% FCS, antibiotic-antimycotic mix, and 2 μg/ml 5-FU. We also created stable cell populations of HT-29/EGFP and HT-29/EGFP/FUR cells expressing a nuclear red fluorescent protein using IncuCyte™ NucLight™ Red Lentivirus Reagent according to manufacturer’s protocol (Essen BioScience, UK), and cells are referred to as HT-29/NLR and FUR/NLR, respectively. All cell lines (including virus-producing cells) were regularly tested for mycoplasma contamination by PCR.

### Immunocytochemistry – Evaluation of cell morphology

Cells were washed with PBS and fixed with 4% PFA for 20 min. After incubation with anti-F-actin rhodamine-labeled antibody (Molecular Probes, USA), nuclei were counterstained with DAPI. Staining patterns were analyzed with Zeiss fluorescent microscope and automated imaging Metafer (MetaSystems GmbH, Germany).

### Immunophenotyping

Following fluorochrome-conjugated antibodies were used for the evaluation of surface markers: CD44-PE, CD24-PE, CD26-PE, CD271-PE, CD133/2-PE (Miltenyi Biotec, Germany); CD166-PE (ALCAM; Immunotech, France); CD274 (PD-L1; Sony Biotechnology, USA); CD44v6-PE (RD Systems, USA); CD184 (CXCR4-PE; eBioscience, USA). Dead cells were excluded from the analysis based on DAPI staining. Cells were analyzed using BD FACSCanto™ II flow cytometer (Beckton Dickinson, USA) equipped with FacsDiva program. FCS Express software was used for the evaluation.

### Cell viability assay

The viability of tumor cells after treatment with the concentration gradient of chemotherapeutics [5-FU; cisplatin (EBEWE Pharma, Austria); oxaliplatin (Oxaliplatin Kabi; Fresenius Kabi Oncology, India); cyclophosphamide (Endoxan; Baxter, Germany); irinotecan (Sigma-Aldrich] for indicative time (6 days) was determined using CellTiter-Glo® Luminescent Cell Viability Assay (Promega Corporation, Madison, WI) performed according to manufacturer’s instructions, and the relative luminescence was evaluated as in [26]. Briefly, the test was performed in 96-well format in quadru- to sextaplicates. Plated tumor cells were let to adhere overnight, and the next day the drugs were added. At the experimental endpoint, the values were expressed as means of replicates ± SD and expressed as percentage of relative viability. The luminescence of control cells without drug was taken as 100% viability. The IC_50_ values were counted using CalcuSyn program.

### Detection of apoptosis by Annexin V assay

Twenty four hours after plating of cells (5 × 10^4^/well in 24-well plates) the chemotherapeutics 5-FU, cisplatin, doxorubicin (EBEWE Pharma), cyclophosphamide, and paclitaxel (Paclitaxel Mylan; Oncotec Pharma Produktion GmbH, Germany) at desired concentrations were added to the respective wells, and cells were treated for 48 h. Harvested cells (also the ones from supernatant) were washed in PBS, then once in Binding Buffer (1 mM HEPES, 14 mM NaCl, 2.5 mM CaCl_2_ in PBS). The pellet was subsequently resuspended in Binding Buffer containing PE-conjugated Annexin V (eBioscience, San Diego, CA). Incubation took 10 min at room temperature, protected from light. DAPI solution (0.1 μg/ml) was added to detect dead cells. Analysis was made by BD FACSCanto™ II flow cytometer equipped with FacsDiva program, and data were analyzed with FCS Express program.

### Immunomagnetic separation

In order to separate the CD133^+^ cell subpopulation from HT-29/EGFP/FUR cultures, the MACS positive cell separation protocol with CD133 MicroBeads (Miltenyi Biotec) and CD133/2-PE antibody (Miltenyi Biotec) was used according to the manufacturer’s recommendation. Briefly, 5–17 × 10^6^ cells were loaded onto a MACS_MS Column (Miltenyi Biotec) placed in the magnetic field of a MACS Separator (Miltenyi Biotec). Flowthrough from the column was collected as the CD133-negative fraction (FUR/CD133-). Columns were washed three times, and then the positive fraction (FUR/CD133+) was eluted, and used in subsequent experiments.

### Expression analysis

Total RNA was isolated from 1-2 × 10^6^ tumor cells by NucleoSpin® RNA II Mini Total RNA Isolation Kit (Macherey Nagel, Germany) according to the protocol. RNA was reverse transcribed with RevertAid™ H minus First Strand cDNA Synthesis Kit (Thermo Scientific, USA). Quantitative PCR was performed in 1× GoTaq® qPCR Master Mix (Promega, Madison, WI, USA) with specific primers (10 pmol/μl) and 1 μl of template cDNA on Bio-Rad CFX96™ Real-Time PCR Detection System (Bio-Rad, USA) using the following protocol: activation step at 95 °C for 2 min, 40 cycles of denaturation at 95 °C for 15 s, 1 min annealing and polymerization at 60 °C with plate read for 5 s at 76 °C followed by denaturation at 95 °C for 10 s, and final extension for 5 s at 65 °C, then melt curve analysis. Obtained data were subsequently analyzed using CFX Manager™ Software (Version 1.5). Gene expression was calculated using delta cycle threshold values (ΔCt = Ct_TARGET GENE_ – Ct_REFERENCE GENE_). The expression of *HPRT1* and *GAPDH* genes was set as an endogenous reference gene. Analysis was performed in quadruplicates and data were expressed as means ± SD. The table of primers sequences used for expression analysis is in Table 1.

### DNA extraction and qPCR analysis for detection of human sequences in mouse lung

Mice lung tissue was mechanically dissociated in the presence of liquid nitrogen. The genomic DNA was isolated by NucleoSpin® Tissue isolation kit (Macherey Nagel) according to the protocol. DNA was analysed for the presence of the mouse *Rapsn* (receptor-associated protein at the synapse) gene and the human *HBB* (β*-*GLOBIN) gene by duplex qPCR as published previously [27]. DNA isolated from human cells HT-29/EGFP/FUR was used as positive control for the presence of *HBB*, DNA from healthy mouse lung was taken as positive control for mouse *Rapsn* gene, and DNA extracted from mouse lung with macroscopically detected and immunohistochemically proven presence of HT-29/EGFP/FUR-induced metastases was used as positive control for both human and mouse sequences. After PCR, 10 μl of PCR products were detected in 4.5% MetaPhor® Agarose (Cambrex, USA) prepared by manufacturer’s instructions.

### Gene expression array

For evaluation of the effect of long-term maintenance of HT-29 colon cancer-derived cells in 5-FU on the expression of specific human genes related to stem-cells, we used the Human Stem Cell RT^2^ Profiler PCR Array (PAHS-405ZA; Qiagen, Germany) profiles in parental and chemoresistant cell lines. RNA from 5 × 10^5^ cells of HT-29/EGFP and HT-29/EGFP/FUR were isolated by AllPrep RNA/Protein kit (Qiagen), and subsequently reverse-transcribed with RT^2^ First Strand Kit (Qiagen). Assay was performed using RT^2^ SYBR Green Mastermix (Qiagen) according to manufacturer’s instructions. The assay was performed on Bio-Rad CFX96™ Real-Time PCR Detection System.

### Evaluation of aldehyde dehydrogenase (ALDH) activity

To evaluate the ALDH activity in tested cell lines, functional ALDEFLUOR assay was performed using ALDEFLUOR™ Kit (StemCell Technologies) according to manufacturer’s instructions. Dead cells were excluded from analysis based on DAPI staining. Measurement was performed using BD FACSCanto™ II flow cytometer equipped with FacsDiva program. Data were analyzed with FCS Express program.

### Western blot

Cells were lysed by using All/Prep RNA/Protein Kit (Qiagen), and proteins were quantified by NanoDrop ND-1000 U*V*/Vis Spectrophotometer (NanoDrop Technologies, USA). Protein samples (5 μg) were loaded onto a 10% polyacrylamide gel. Rabbit Anti-ALDH1A3 (Abcam) was used for detection, monoclonal Anti-β-Actin (Sigma-Aldrich) served as a loading control. Secondary HRP-conjugated antibodies were used - anti-rabbit IgG, HRP-linked Ab (Cell Signaling Technology) for ALDH1A3, and anti-mouse IgG, HRP-linked Ab (Cell Signaling Technology) for β-actin. Immunoblots were visualized using enhanced chemiluminescence (Bio-Rad, Clarity™ Western ECL Substrate).

### Gene expression silencing by siRNA interference

Suspension of 1-2 × 10^6^ HT-29/EGFP/FUR cells was transfected with small-interfering RNA (siRNA) oligonucleotides according to the manufacturer’s instructions using the Neon® transfection system (Invitrogen, USA). The following electroporation parameters were used: pulse voltage 1400 V; pulse width 20 ms; pulse number 1. The siRNA oligonucleotides were purchased from Sigma-Aldrich: ALDH1A3 (MISSION® siRNA SASI_Hs01_00129096 and MISSION® siRNA SASI_Hs01_00129097 mixed 1:1 to reach efficient silencing of *ALDH1A3* gene), and negative control (SIC001-10NMOL, MISSION® siRNA Universal Negative Control 1). After cultivation for 24, 48 or 72 h, cells were harvested and used for subsequent experiments.

### In vivo experiments

Six to eight-weeks-old male SCID/bg mice (Charles River, Germany) were used in accordance with the institutional guidelines under the approved protocols. Project was approved by the Institutional Ethic Committee and by the national competence authority (State Veterinary and Food Administration of the Slovak Republic), registration No. Ro 2807/12–221 in compliance with the Directive 2010/63/EU and the Regulation 377/2012 on the protection of animals used for scientific purposes. It was performed in the approved animal facility (license No. SK PC 14011). Bilateral subcutaneous xenografts (*n* = 8) in SCID/bg mice were induced by 2 × 10^6^ HT-29/EGFP/FUR cells resuspended in 150 μl serum-free DMEM diluted 1:1 with ECM gel (extracellular matrix; Sigma-Aldrich). Mice were sacrificed 5 months after the tumor cell administration. One small s.c. tumor was cut into small pieces and seeded in culture medium for tumor-cell expansion. In vivo-derived cell line (hereafter referred to as FURiv) was established and used for subsequent experiments. Lung tissues were analyzed by histology and immunohistochemistry as stated below. Alternatively, another seven SCID/bg mice were s.c. injected bilaterally with 2 × 10^6^ HT-29/EGFP/FUR cells as stated above, and at the experimental endpoint (4–5 months after injection) s.c. tumors along with lung tissue were dissociated to single-cell suspensions according to Tumor Dissociation Kit (MACS Miltenyi Biotec) with subsequent G418 selection for establishing spontaneous metastatic tumor cells for in vitro culture. DNA isolated from these lungs was inspected for the presence of human sequences also by molecular analysis as described above.

In order to test the capability of chemonaïve HT-29/EGFP cells to give rise to spontaneous metastatic foci in lungs, we subcutaneously injected mice (*n* = 7) with 2 × 10^5^ cells resuspended in 150 μl serum-free DMEM diluted 1:1 with ECM gel. At the experimental endpoint one month after cells’ injection, lungs were processed for molecular analysis as described above.

### Histology and immunohistochemistry

Formalin fixed, paraffin embedded lung and subcutaneous tumor tissues were cut into 5 μm thick sections, stained with hematoxylin/eosin and immunohistochemically stained for EGFP detection of tumor cells as in [27].

### Wound healing assay

One hundred thousand HT-29/EGFP and 65,000 HT-29/EGFP/FUR cells per well were plated in quadruplicates on ImageLock 96-well plates (Essen BioScience, UK), and let to adhere for 24 h. Confluent monolayers were wounded with wound making tool (IncuCyte WoundMaker; Essen BioScience), washed twice and supplemented with fresh culture medium. Images were taken every 2 h for the next 48 h in the IncuCyte ZOOM™ kinetic imaging system (Essen BioScience). Cell migration was evaluated by IncuCyte ZOOM™ 2016A software based on the relative wound density measurements and expressed as means of two independent experiments run in quadruplicates ± SD.

### Statistical analysis

All experiments were performed at least trice, and representative results were shown. Data involving comparison between two groups were analyzed by either unpaired Student’s t-test or nonparametric Mann-Whitney U test. Comparison of more than two groups was analyzed by nonparametric Kruskal-Wallis test. The data were analyzed by GraphPad Prism® software (LA Jolla, CA, USA). The value of *p* < 0.05 was considered statistically significant (*p < 0.05; **p < 0.01; ***p < 0.001, ****p < 0.0001).

## Results

### Acquired resistance is associated with multiple alterations in HT-29/EGFP/FUR cells

**Fig. 1:**
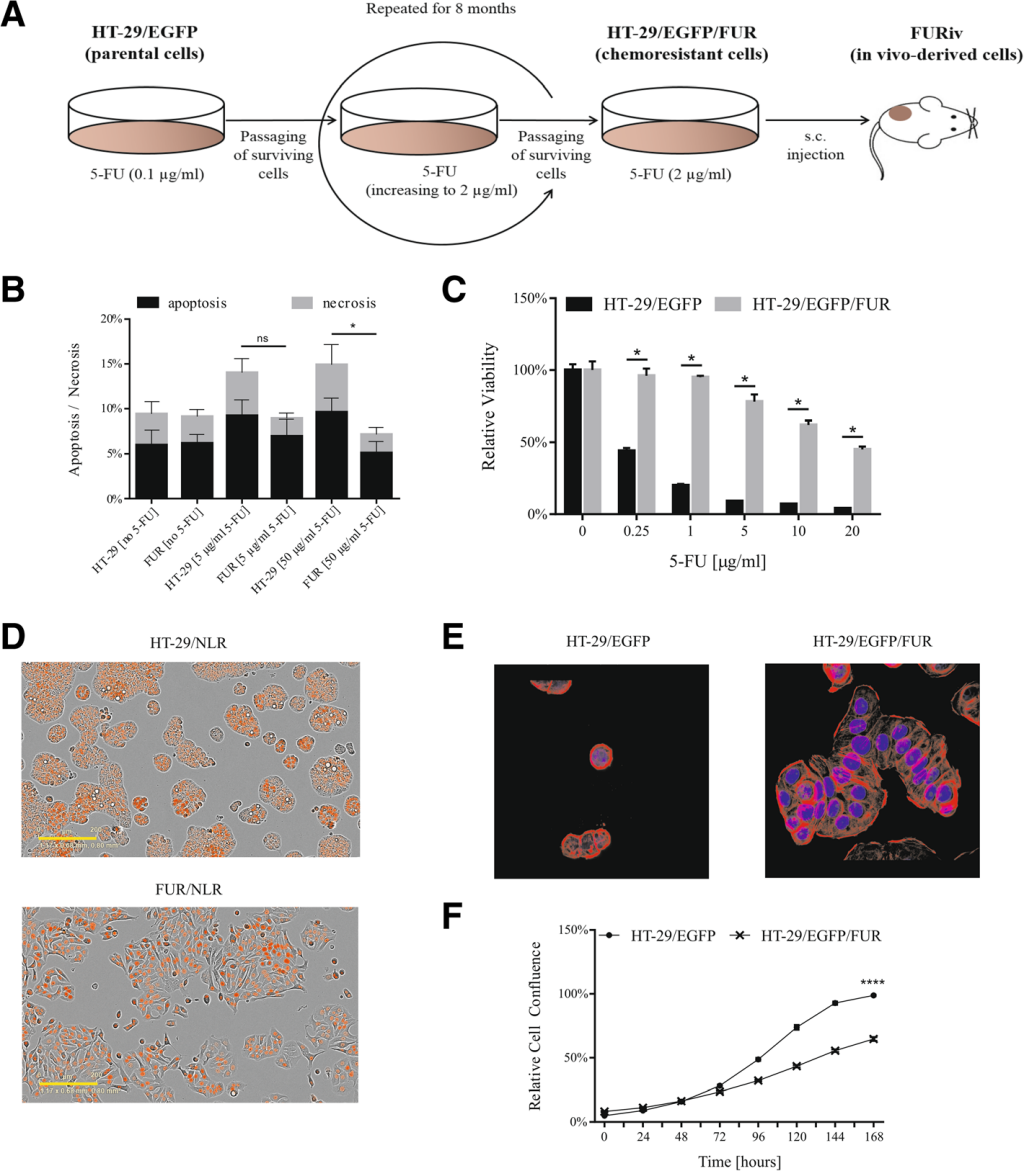
HT-29/EGFP/FUR cells are resistant to 5-FU, exhibit altered morphology and decreased proliferation. **a** HT-29/EGFP/FUR cells stably resistant to 5-FU were developed by exposing cell line HT-29/EGFP to gradually increasing concentrations of this chemotherapeutic till stable proliferation in concentration of 2 μg/ml. The chemoresistant cells, and in vivo-derived (FURiv) cells were used for subsequent analysis. **b** The chemoresistant cells (here referred to as FUR) exerted reduced apoptosis. The decreased proportion of apoptotic cells was determined by Annexin V assay. Cells positive for Annexin V are apoptotic, positivity for DAPI stands for necrotic ones. Mann-Whitney U test was used for statistical analysis. **c** Chemoresistant cells are 135-fold more resistant to 5-FU than parental counterparts HT-29/EGFP as evaluated by luminescence viability assay after 6-days-long treatment. Values were expressed as means of quadruplicates ± SD. Mann-Whitney U test was used for statistical analysis. **d** Fluorescent images from IncuCyte ZOOM™ kinetic imaging system documented altered cellular morphology and formation of colonies of chemoresistant cells stained with NucLight™ Red (referred to as FUR/NLR) when compared to parental cell line (also stained with red nuclei protein; HT-29/NLR). Scale bar: 200 μm. **e** Immunocytochemical staining of F-actin showed morphological differences between chemoresistant and parental cells. Magnification × 630. **f** Based on counting of the confluence by the kinetic imaging system we have shown that the proliferation rate of HT-29/EGFP cells and their chemoresistant counterparts significantly differ. SD are below resolution of the picture. Student’s t-test was used for the statistical analysis

**Fig. 2:**
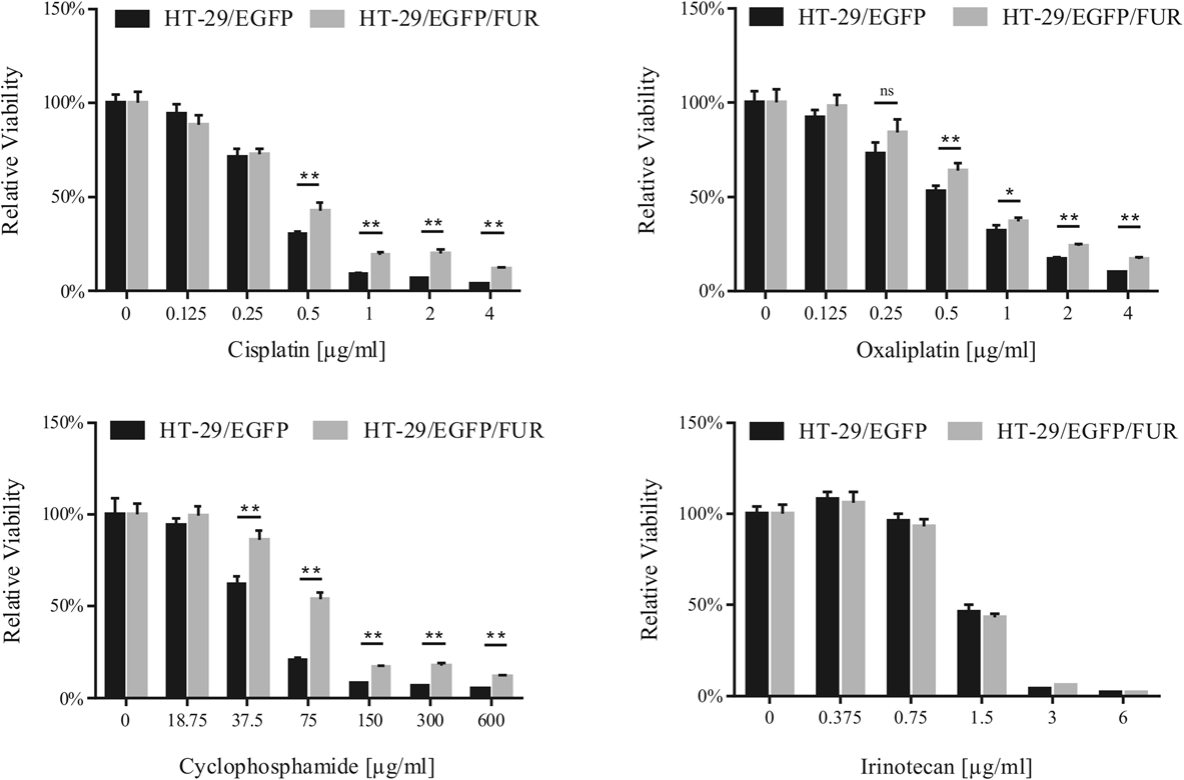
The chemoresistant HT-29/EGFP/FUR cells are cross-resistant to cisplatin, oxaliplatin and cyclophosphamide. HT-29/EGFP/FUR cells exert cross-resistance to chemotherapeutics with different mechanism of action - cisplatin, oxaliplatin, cyclophosphamide as evaluated by the luminescence viability assay performed after 6 days of the cultivation in the presence of drugs. Values were expressed as means of sextaplicates ± SD. Mann-Whitney U test was used for statistical analysis

We derived chemoresistant cell line variant HT-29/EGFP/FUR by continuous culture of HT-29/EGFP cells in increasing concentration of 5-FU, and selection of surviving cells for 8 months. We declared these cells chemoresistant after they achieved ability to stably proliferate in the clinically relevant plasma concentration of 5-FU (2 μg/ml) in which they have still been maintained since then (Fig. 1a). Increased resistance to 5-FU was demonstrated by Annexin V assay after 48 h-treatment by 5-FU. We detected almost two-fold decreased proportion of HT-29/EGFP/FUR apoptotic cells in comparison to HT-29/EGFP (Fig. 1b). In order to compare the response of HT-29/EGFP and its chemoresistant counterparts to 5-FU, we also used the luminescent assay based on ATP quantitation representative of metabolically active cells. Direct comparison of the sensitivity to 5-FU (after 6 days-long cultivation) revealed shift in IC_50_ values making HT-29/EGFP/FUR cells 135-fold more resistant over parental counterparts (Fig. 1c; Table 2). We observed that stable maintenance of cells in the presence of 5-FU increased the resistance gradually with the increasing number of passages (data not shown). In comparison to parental cells, HT-29/EGFP/FUR cells formed bigger and more spread colonies (Fig. 1d) and changed their morphology (Fig. 1e). In order to provide kinetic measurements, we stained tumor cells with red nuclear protein (NLR). Based on confluence evaluation in IncuCyte ZOOM™ kinetic imaging system we observed that resistant cells exhibit a significant decrease in cellular proliferation in vitro (Fig. 1f). Of note, we observed that chemoresistant cells in low passages were more quiescent, based on the doubling time calculations. The doubling time of parental HT-29/EGFP cells was 29 h, whereas the doubling times of low-passaged HT-29/EGFP/FUR cells was 51 h and for high-passaged cells the doubling time shortened to 40 h after overcoming this intermittent step. The evaluation of sensitivity of chemoresistant cells to panel of drugs with different mechanism of action proved that long-term cultivation of HT-29/EGFP cells in 5-FU-containing medium led to cross-resistance to cisplatin, oxaliplatin, cyclophosphamide, and no difference in sensitivity to irinotecan, as quantified by luminescent viability assay (Fig. 2) with counted IC_50_ values (Table 3).

**Table 1:** Sequences of primers used for expression analysis

Gene	Product size	Forward primer (5` to 3`)	Reverse primer (5` to 3`)
*HPRT1*	137 bp	TGACCAGTCAACAGGGGACA	ACTGCCTGACCAAGGAAAGC
*GAPDH*	226 bp	GAAGGTGAAGGTCGGAGTC	GAAGATGGTGATGGGATTTC
*ALDH1A3*	133 bp	GCCCTTTATCTCGGCTCTCT	CGGTGAAGGCGATCTTGT
*ALDH2*	168 bp	ACCTGGTGGATTTGGACATGGTCC	TCAGGAGCGGGAAATTCCACGGA
*ALDH1A2*	193 bp	AGGGCAGTTCTTGCAACCATGGAA	CACACACTCCAATGGGTTCATGTC
*ALDH3A1*	192 bp	TGTGTCAAAGGCGCCATGAGCAAG	GGCGTTCCATTCATTCTTGTGCAG
*ALDH1A1*	182 bp	TTGGAATTTCCCGTTGGTTA	CTGTAGGCCCATAACCAGGA

**Table 2:** IC_50_ values for 5-fluorouracil

	IC_50 5-FU_ [μg/ml]
HT-29/EGFP	0.15 (0.12–0.17) *r* = 0.96
HT-29/EGFP/FUR	20.2 (10.5–38.7) *r* = 0.97

**Table 3:** IC_50_ values of cisplatin (CisPt), oxaliplatin (Ox), irinotecan (IRI) and cyclophosphamide (CPX)

	IC_50 CisPt_ [μg/ml]	IC_50 Ox_ [μg/ml]	IC_50 IRI_ [μg/ml]	IC_50CPX_ [μg/ml]
HT-29/EGFP	0.42 (0.27–0.67) r = 0.96	0.62 (0.52–0.75) *r* = 0.99	1.59 (1.18–2.13) r = 0.97	56.44 (33.89–94.00) *r* = 0.93
HT-29/EGFP/FUR	0.52 (0.34–0.80) r = 0.96	0.97 (0.69–1.36) r = 0.96	1.51 (1.22–1.86) *r* = 0.98	121.22 (76.9–191.2) r = 0.93

**Fig. 3:**
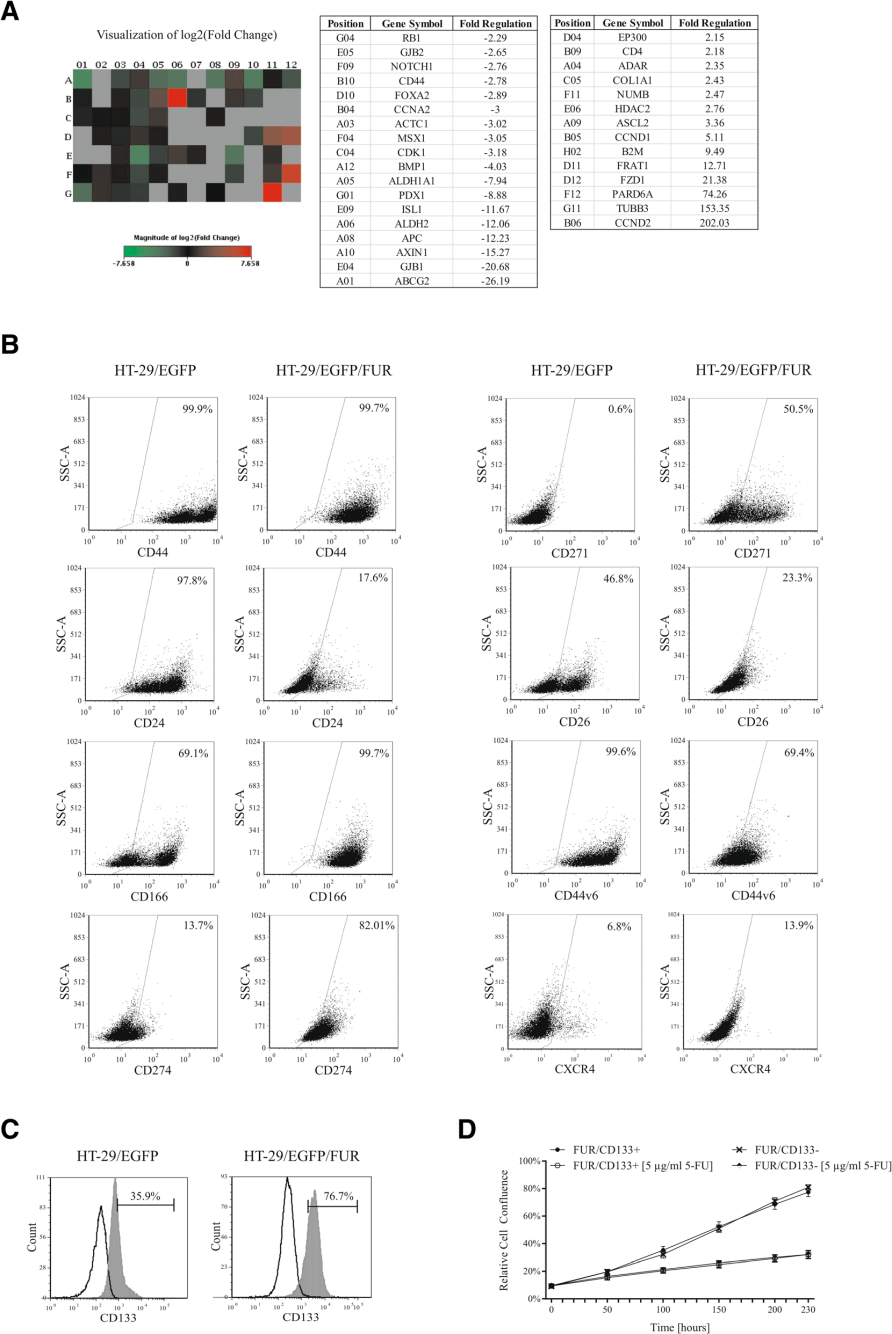
Multiple CSC markers are altered in HT-29/EGFP/FUR cells. **a** The Human Stem Cell RT^2^ Profiler PCR gene expression array revealed statistically significant down- and upregulation of several genes (shown in the table) in HT-29/EGFP/FUR compared to parental HT-29/EGFP cells. **b** The cells were stained with specific anti-human antibodies and analyzed by flow cytometry. The results demonstrated that the chemoresistant cell line was both enriched and depleted for cells expressing specific CSC markers when compared to parental cell line. Isotype control antibodies were used as staining controls. **c** The flow cytometric analysis showed increased expression of CD133 marker in chemoresistant cells over parental counterparts. Black line – isotype control, gray fill – CD133-positive cell population. **d** CD133^+^ and CD133^−^ subpopulations of the chemoresistant cells (referred to as FUR/CD133^+^ and FUR/CD133^−^, respectively) were immunomagnetically separated from bulk HT-29/EGFP/FUR. The proliferation of subpopulations with addition of chemotherapeutic drugs (5-FU depicted as one example) was monitored by the kinetic imaging system. Data demonstrated the same proliferation rates. Values were expressed as means of quadruplicates ± SD

As we built our study on assumption, that CSC and chemoresistant cell subpopulation overlap, we further examined the possibility, that by the long term 5-FU exposure we were able to enrich for the CRC cells with CSC properties. We performed the Human Stem Cell RT^2^ Profiler PCR Array profiles in parental and chemoresistant cell lines. Gene expression array revealed statistically significant downregulation of 18 genes (e.g. ATP Binding Cassette Subfamily G Member 2, *ABCG2*; Aldehyde Dehydrogenase 2, *ALDH2*; Axin-1, *AXIN1*; Adenomatous Polyposis Coli, *APC*; Cyclin Dependent Kinase 1, *CDK1*; notch 1, *NOTCH1*), and upregulation of 14 genes (e.g. cyclin D2, *CCND2*; Tubulin Beta 3 Class III, *TUBB3*; Frizzled Class Receptor 1, *FZD1*; achaete-scute family bHLH transcription factor 2, *ASCL2*) in HT-29/EGFP/FUR compared to parental cells HT-29 as shown in table (Fig. 3a).

Flow cytometric analysis (Fig. 3b) of stem cell-associated markers showed that chemoresistant cell line HT-29/EGFP/FUR has no changes in total number of CD44-positive cells. Surprisingly, we observed decreased number of cells positive for CD44v6, CD24, and CD26. On the other hand, chemoresistant cell line was enriched for cells that express CSC marker CD166 (99.7% of cells relative to 69.1% of parental cells), CD274 (also referred to as PD-L1; 82% of cells relative to 13.7% of parental cells). Surprisingly, the expression of CD271, the nerve growth factor receptor, was induced in these cells (50.5% of cells compared to no expression in parental cells). In our experiments, chemonaïve cells had 6.8%-positivity of CXCR4, chemoresistant cells were assigned with 13.9%-positivity. Flow cytometry revealed also overexpression of CSC marker CD133 in HT-29/EGFP/FUR in comparison to their parental counterparts (76.7% of chemoresistant cells relative to 35.9% of HT-29 cells; Fig. 3c), and we wanted to determine if this subpopulation might be responsible for resistance to chemotherapeutics. Immunomagnetically separated HT-29/EGFP/FUR-CD133^+^ cell fraction (referred to as FUR/CD133+) represented 22.05% (range 4.5–36.8%, number of isolations *n* = 4) of all cells. FUR/CD133^+^ subpopulation exhibited similar viability (no change in IC_50_ values) and no difference in proliferation rate when treated with 5-FU, doxorubicin, cisplatin, and cyclophosphamide in comparison to CD133^−^ fraction as shown by kinetic imaging system (Fig. 3d depicted as one example).

In summary, these data depict that chemoresistant cells derived from HT-29 cell line show multiple alterations in morphology, exhibit decreased proliferation, cross-resistance and multiple alterations in expression of CSC markers. Moreover, we confirmed that CD133 subpopulation was not responsible for chemoresistance in this model.

### Chemoresistant colon cancer cells form spontaneous metastases and retained their resistance in vivo

**Fig. 4:**
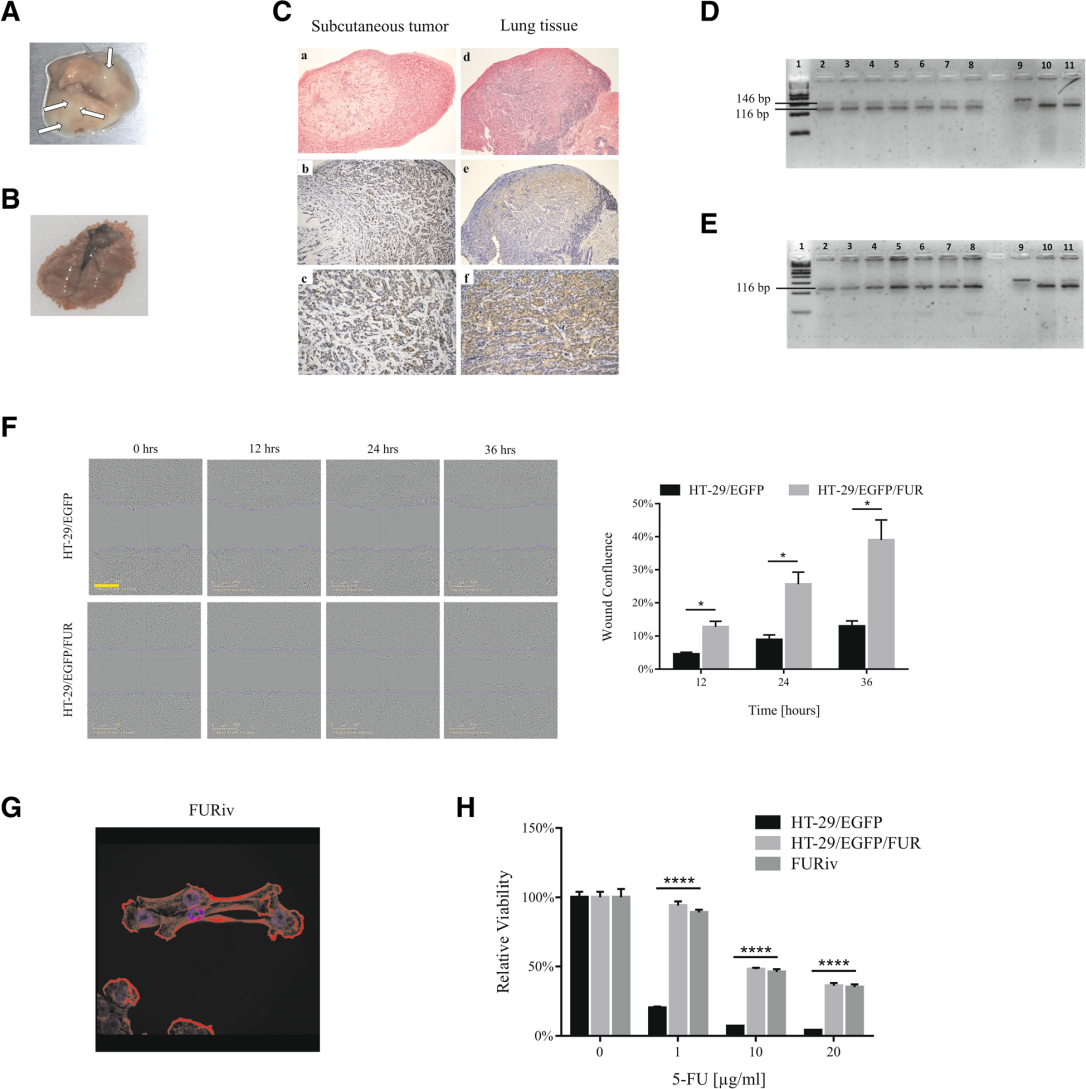
The chemoresistant HT-29/EGFP/FUR cells spontaneously form lung metastases whilst retaining chemoresistance. **a** The lung inspection unraveled multiple tumor metastasis foci in the lung parenchyma of the animals subcutaneously injected with HT-29/EGFP/FUR cells in comparison to the lung of mice injected with chemonaïve HT-29/EGFP cells (**b**). **c** The histological and immunohistochemical analysis of mouse tissues revealed the lung metastases development five months after subcutaneous administration of HT-29/EGFP/FUR cells. **a** Subcutaneous tumor. Hematoxylin/eosin staining, original magnification × 100. **b**, **c** Detection of EGFP-expressing tumor cells in subcutaneous tumor. Immunohistochemical reaction with anti-EGFP polyclonal antibody, original magnification × 200, × 400, visualization with 3,3′-diaminobenzidine. **d** Lung tumor. Hematoxylin/eosin staining, original magnification × 100. **e**, **f** Detection of EGFP-expressing tumor cells in lung tumor. Immunohistochemical reaction with anti-EGFP polyclonal antibody. Original magnification × 100, × 400, visualization with 3,3′-diaminobenzidine. **d** The molecular analysis of lungs of mice (*n* = 7) injected with HT-29/EGFP/FUR cells with detected metastases. DNA was PCR amplified for human and mouse gene detection. 1. Molecular weight standard (50 bp). 2. – 8. Product of duplex PCR - seven mouse lungs. Both human *HBB* (146 bp) and mouse *Rapsn* (116 bp) are present. 9. Positive control for human DNA. 10. Positive control for mouse DNA. 11. Positive control for both human and mouse DNA. On the contrary, we did not detect the presence of human sequences in lungs of mice (n = 7) injected with chemonaïve HT-29/EGFP cells (**e**; lines 2. – 8.). **f** The representative images taken at 12, 24 and 36 h after the monolayer wounding exhibit higher cell confluence in the wounded area of HT-29/EGFP/FUR cells. Blue lines indicate the initial scratch wound border, scale bar: 400 μm. The chemoresistant cells significantly increased the migration as determined by higher relative wound confluence. Mann-Whitney U test was used for statistical analysis. **g** The immunocytochemical staining of F-actin showed dramatic morphological difference of in vivo-derived HT-29/EGFP/FUR (referred to as FURiv) cells to fibroblastoid-like shape. Magnification × 630. **h** FURiv cells retained their resistance even after 5-months-long in vivo growth, and subsequent 2-months-long in vitro cultivation without the selection pressure of 5-FU. The cells were treated with different doses of 5-FU for 6 days, and the viability was evaluated by the luminescence assay. The sensitivity to 5-FU was exactly the same as for HT-29/EGFP/FUR cells from which the xenotransplant and subsequent FURiv-derivative had been established. Kruskal-Wallis test was used for statistical analysis

Two millions of HT-29/EGFP/FUR cells were subcutaneously injected in SCID/bg mice (*n* = 8 xenografts) to determine their tumorigenic potential. The tumor growth was very slow, 7 xenografts regressed within one to two months. Only one xenograft was developed not exceeding volume of 40 mm^3^ during five months. At the experimental endpoint, five months after inoculation, mice were sacrificed. Macroscopically visible metastases were detected in lung of one animal (Fig. 4a). On contrary, no visible metastases were seen in lung from mice injected with chemonaïve HT-29/EGFP cells (Fig. 4b). Histological (hematoxylin/eosin) staining confirmed presence of tumor cells in lung injected with chemoresistant cells, and immunohistochemical (EGFP detection) analysis proved their origin in HT-29/EGFP/FUR cells (Fig. 4c). Chemoresistant cell line HT-29/EGFP/FUR is thus aggressive enough to be able to induce lung metastases even after subcutaneous administration. The presence of metastases was massive - tumor lesions were presented across the whole lung tissue. To confirm this result, another seven SCID/bg mice were subcutaneously injected with chemoresistant cells. Subcutaneous tumors became palpable between 49th to 124th day after inoculation in all animals. Chemoresistant tumor cells were detected in lungs of these seven animals by qPCR as molecular analysis confirmed human *HBB* specific sequences in all animals (Fig. 4d) in contrast to no presence of human sequences in mice (*n* = 7) injected with HT-29/EGFP cells (Fig. 4e). Lungs with macroscopic tumor lesions of HT-29/EGFP/FUR cells from sacrificed mice (4–5 months after inoculation) were dissociated into single-cell suspensions and expanded in vitro after G418 selection. We isolated and expanded metastatic cells from lungs from five out of seven mice (71.4%).

Based on this result, we assumed that the metastatic capability might be associated with differential migratory potential of chemoresistant cells. Using the scratch wound assay, we evaluated the migration potential of chemoresistant cells in comparison to their parental counterparts. The migration of HT-29/EGFP/FUR was clearly evident from the beginning where 100% confluent monolayer region of cells moved into the cell-free scratch region. The wound confluence was quantified by using IncuCyte ZOOM™ kinetic imaging system. We showed that the migration ability of chemoresistant cells is three-fold higher (25.6% vs 8.9% after 24 h) in comparison to HT-29/EGFP cells (Fig. 4f).

In order to analyze the properties of the chemoresistant cells after in vivo growth, we also expanded cells from small xenotransplant of subcutaneously injected HT-29/EGFP/FUR cells. The cell line was established, and was referred to as FURiv cells (in vivo-derived cells). The morphology of these cells changed dramatically to more fibroblastoid-like in comparison to in vitro chemoresistant cells (Fig. 4g). The luminescent viability assay measured at day 6 after the start of the treatment with 5-FU showed that FURiv cells retained their resistance even after 5-months-long in vivo growth, and subsequent 2-months-long cultivation in vitro without the selection pressure of the drug. The sensitivity to 5-FU was exactly the same as for HT-29/EGFP/FUR cells from which the xenotransplant and subsequent FURiv-derivative had been established (Fig. 4h).

In summary of this part, we demonstrated unexpected association between chemoresistance and metastatic capabilities of the prepared cell line variant. Chemoresistant cells exhibited increased migratory potential, very low tumorigenicity upon s.c injection, however, they can survive in vivo for a long time and produce distant metastases.

### Increased chemoresistance correlates with ALDH activity and it can be reverted by ALDH1A3 knockdown

**Fig. 5:**
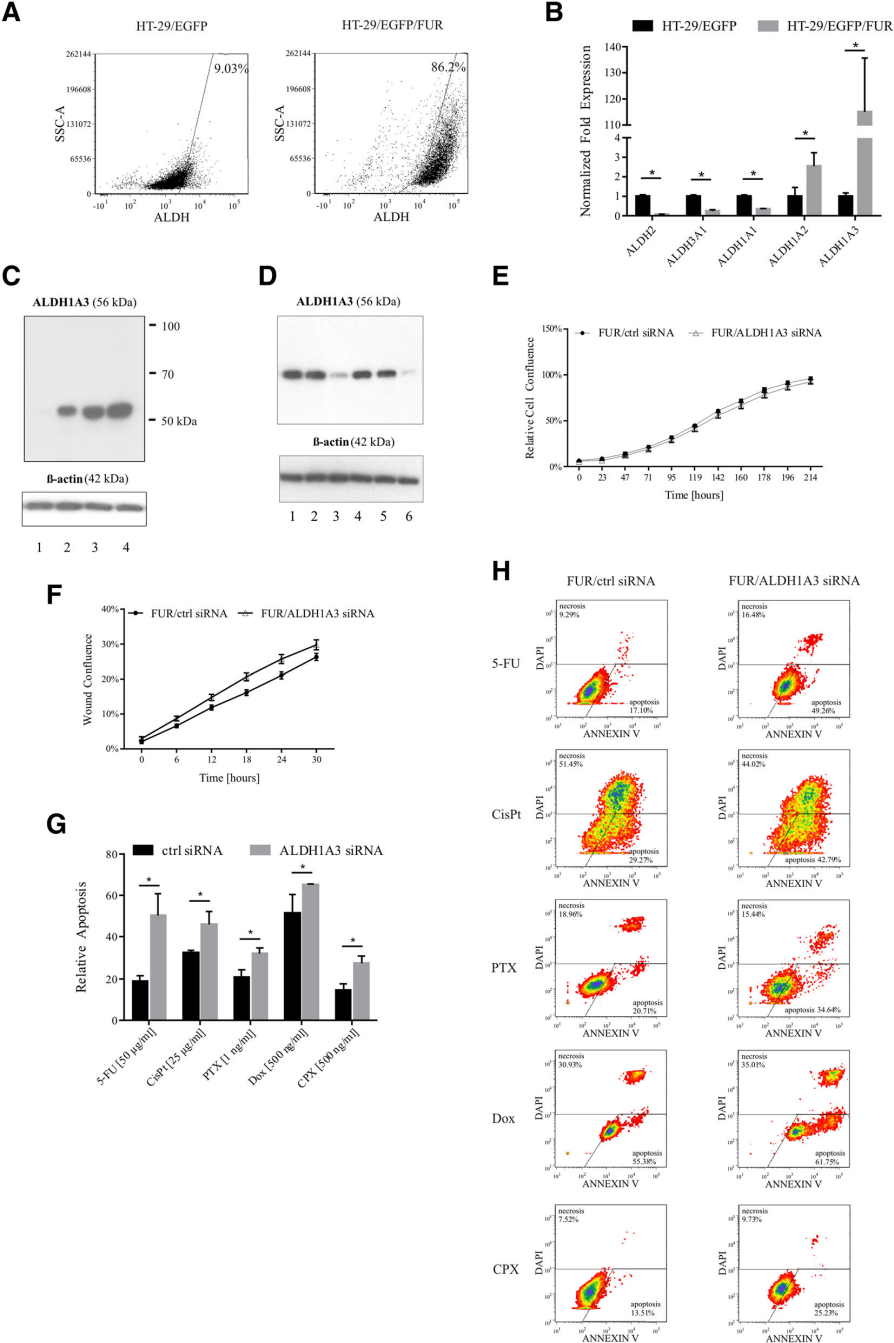
The increased ALDH activity correlated with chemoresistance and *ALDH1A3* silencing partially reverted the resistance. **a** The flow cytometry analysis by Aldefluor Assay revealed 9.5-fold increased ALDH activity in chemoresistant cells when compared to chemonaïve counterparts. **b** The chemoresistant cells switched the expression of ALDH isoforms as demonstrated by qPCR. The expression in parental HT-29/EGFP cells was set as a reference, and *GAPDH* and *HPRT1* served as an internal control. The data are expressed as means of quadruplicates ± SD. Mann-Whitney U test was used for statistical analysis. **c** The Western blot analysis confirmed increased expression of ALDH1A3 also on the protein level. The longer cultivation of chemoresistant cells in the medium with 5-FU, the higher amount of protein. 1. HT-29/EGFP; 2. HT-29/EGFP/FUR low passage; 3. HT-29/EGFP/FUR middle passage; 4. HT-29/EGFP/FUR high passage. β-actin was used as an internal loading control. **d** The Western blot analysis after RNA silencing proved successful *ALDH1A3* inhibition as shown 48 and 72 h post nucleofection. 1. HT-29/EGFP/FUR no treatment at 48 h; 2. FUR/ctrl siRNA at 48 h; 3. FUR/ALDH1A3 siRNA at 48 h; 4. HT-29/EGFP/FUR no treatment at 72 h; 5. FUR/ctrl siRNA at 72 h; 6. FUR/ALDH1A3 siRNA at 72 h. β-actin was used as an internal loading control. **e** The molecular silencing of *ALDH1A3* by specific siRNA did not affect the proliferation rate of chemoresistant cells (referred to as FUR/ALDH1A3 siRNA) as detected by the kinetic imaging system. The control siRNA was used as a negative control (FUR/ctrl siRNA). **f** The molecular *ALDH1A3* silencing did not either affect the migration potential of chemoresistant cells as determined by the wound healing assay. FUR/ALDH1A3 siRNA-cells filled in 29% of wound in comparison to cells silenced with control siRNA (FUR/ctrl siRNA) which filled in 26% of the wound. **g-h** The silencing of *ALDH1A3* partially reversed chemoresistance of HT-29/EGFP/FUR cells as demonstrated by flow cytometry. The increased sensitivity to chemotherapeutics added 48 h post nucleofection (5-FU, CisPt - cisplatin, PTX - paclitaxel, Dox - doxorubicin, CPX - cyclophosphamide) was induced by the molecular inhibition of *ALDH1A3*. The apoptosis was subsequently determined 48 h later by the Annexin V assay. **g** The data demonstrating apoptosis are expressed as means of quadruplicates ± SD. Mann-Whitney U test was used for statistical analysis. **h** Density plots demonstrating cell death. The gates were defined based on the cells nucleofected by control siRNA without chemotherapeutics. The cells positive for Annexin V are apoptotic, positivity for DAPI stands for necrotic ones

Next, we focused on another potential mechanism contributing to both stemness and drug resistance as described previously. The analysis by functional Aldefluor assay showed changes in ALDH activity of tumor cells long-term cultivated in the presence of 5-FU (86%-positivity of HT-29/EGFP/FUR cells compared to 9% in HT-29/EGFP; Fig. 5a). Detailed analysis of the expression of ALDH isoforms showed that the long-term maintenance of HT-29 colorectal cancer cells in 5-FU resulted in the switch in expression of particular isoforms (Fig. 5b). Quantitative PCR revealed that chemonaïve cells have high expression of ALDH1A1, ALDH3A1 and ALDH2. On the other hand, HT-29/EGFP/FUR chemoresistant cells overexpress ALDH1A2 and ALDH1A3. Western blot analysis confirmed the expression of ALDH1A3 marker also on the protein level on the contrary to no expression in HT-29/EGFP cells. More importantly, we were able to show, that the upregulation of this ALDH isoform was gradual along with the process of chemoresistant cell derivation and propagation (Fig. 5c).

Molecular silencing of ALDH1A3 mRNA led to decreased ALDH activity in general (80.4% ALDH positive cells in control siRNA-treated HT-29/EGFP/FUR cells in comparison to 24.4%-positivity 48 h after inhibition and 10.74%-positivity another 24 h later as evaluated by Aldefluor assay). The RNA interference was confirmed also by qPCR and western blotting (Fig. 5d), and was confirmed also 8 days after nucleofection. The silencing of ALDH1A3 affected neither the proliferation rate of chemoresistant cells (Fig. 5e), nor the migration of chemoresistant cells (Fig. 5f). The ALDH1A3 silencing significantly decreased chemoresistance of HT-29/EGFP/FUR cells to multiple agents. Cells 48 h post transfection were treated with chemotherapeutic drugs 5-FU, cisplatin, paclitaxel, doxorubicin and cyclophosphamide, and the effect was determined 48 h later by flow cytometric Annexin V assay. We observed increased apoptosis in all tested drugs (Fig. 5g-h).

In conclusion, here we describe the novel variant of chemoresistant HT-29 cells with spontaneous metastatic potential. Moreover, we have shown, that single drug exposure resulted in acquired resistance to multiple drugs, which could be partially reverted by silencing of ALDH.

## Discussion

Based on the clinical significance of chemoresistance with increasing number of evidence that tumor cells resistant to chemotherapy represent aggressive subpopulation of cells that could also be leading to metastatic dissemination and relapse of the disease, we prepared cell line derived from HT-29 resistant to 5-FU at the clinically relevant plasma concentration of patients treated by this drug according to [25], and these cells are hereafter referred to as HT-29/EGFP/FUR.

Cells maintained in 5-FU till approximately 30th passage went through “crisis” – they were highly vacuolised with low proliferation rate and changed morphology. After the cells overcame this “crisis”, the proliferation rate increased, and the morphology changed, too. Also in accordance with [25], chemoresistant derivative exerts cross-resistance which suggests that acquired resistance to one chemotherapeutic activates general resistance pathways that impart resistance to multiple agents. The expression profile array (Fig. 3a) showed upregulation of genes in HT-29/EGFP/FUR cells which for example were shown to play part in migration, invasion and correlated with aggressive prostate cancer features - par-6 family cell polarity regulator alpha, *PARD6A* [28]; were proven to be unfavorable prognostic marker – Cyclin D1, *CCND1* [29]; were a cellular survival factor and biomarker of poor outcome – Tubulin Beta 3 Class III, *TUBB3* [30]; were a putative tumor promoter – Cyclin D2, *CCND2* [31]. It was demonstrated [14, 32] that high Wnt activity functionally designates the colon cancer stem population. In normal cells, the transcriptional regulator β-catenin is tightly controlled by a multiprotein complex that contains the tumor suppressor Adenomatous Polyposis Coli (APC). Activation of Frizzled receptors by Wnt ligands disrupts this complex and results in the translocation of β-catenin to the nucleus, and the expression of Wnt target genes. Thanks to high overexpression of Frizzled Class Receptor 1 *(FZD1*) - a receptor for Wnt proteins, and *FRAT1* - a positive regulator of this pathway, along with downregulation of APC and Axin-1 (*AXIN1*) - a negative regulator of Wnt signaling pathway, we can assume that Wnt signaling cascade could have been activated in HT-29/EGFP/FUR cells, and thus further experiments clarifying the role of Wnt signaling in this chemoresistant cell line are needed. It is of our interest to define its activity because Wnt signaling was proven to be involved in epithelial-to-mesenchymal transition (EMT) playing part in migration process [33].

Long-term cultivation of cells in 5-FU led to changed expression of cell-surface markers whose roles were implicated in stem cell-like behavior. Chemoresistant cells started expressing CD271, which was described as a crucial determinant of tumorigenicity, stem-like properties, heterogeneity and plasticity in melanoma [34], in contrast to no expression in chemonaïve HT-29 cells. CD133 and CD26 have been identified as markers of long-term growth and resistance in HCT-116 colon cancer cell line [35], and CD133 subpopulation itself was demonstrated to induce tumors in mice that resembled the original malignancy [36, 37]. HT-29/EGFP/FUR cells increased expression of CD133 marker, but we detected that mentioned subpopulation positive for CD133 is not assigned with increased resistance to chemotherapeutics over cells negative to this marker. This result is not in conformity with published data claiming that CD133^+^ cells are chemoresistant to 5-FU through the mechanism which is related with survivin expression [38]. Increased expression of CXCR4 in HT-29/EGFP/FUR cells is in conformity with published data proving that CD133^+^CXCR4^+^ cancer stem cells were necessary for tumor metastasis in pancreatic cancer [39] as well as CXCR4 role in oxaliplatin-resistant colorectal cancer cells [5]. Expression of CD166 in HT-29/EGFP/FUR cells contributes to aggressiveness as supported by previous studies [40, 41]. We showed that chemoresistant cells have decreased potential to proliferate in vivo over parental cells HT-29/EGFP. Two millions of subcutaneously injected cells in immunodeficient mice resulted in slow tumor progression. On the contrary, 2.5 × 10^5^ HT-29/EGFP cells are sufficient for quick exponential tumor growth [42]. Our long waiting for tumor growth with subsequent tissue analyses after the end of experiment (5 months after cell injection) let us reveal that chemoresistant cells are spontaneously metastatic - subcutaneous injection of cells resulted in massive lung metastases formation (Fig. 4a, c, d). We associate the acquired metastatic potential of HT-29 cells long-term cultivated under 5-FU pressure to the presence of subpopulations positive for CD133, CD26, CD44v6, CXCR4 and 6-fold higher expression of CD274 along with high overexpression of *PARD6A* and FGF1, FGF2, B2M – markers previously described as triggers of metastasis induction [12, 13, 28, 39, 43–49]. We also showed three-fold higher migration ability of chemoresistant cells in comparison to their parental counterparts, which is in accordance with [5].

Increased ALDH activity in our chemoresistant derivative indicates that long-term maintenance of HT-29 colon cancer cells in 5-FU increased population of cancer stem-like cells. To our knowledge, such a study determining the role of ALDH1A3 in colon cancer model has not been conducted so far. Silencing of high overexpression of ALDH1A3 in HT-29/EGFP/FUR cells (compared to parental counterparts) let to decreased ALDH activity in general, and even though the proliferation rate and migration was not affected, it significantly increased proportion of apoptotic cells treated with panel of drugs (Fig. 5g-h). Chen an colleagues [50] found that knockdown of ALDH1A3 expression in human cholangiocarcinoma cell lines markedly reduced not only their sensitivity to gemcitabine, but also their migration, and most importantly, this enzyme was also identified as an independent poor prognostic factor for patients with intrahepatic cholangiocarcinoma, as well as a prognostic biomarker of gemcitabine-treated patients.

## Conclusions

Our study demonstrated that the chemoresistant population, which can be selected during chemotherapy, leads to metastasition and overexpression of ALDH1A3 isoform in HT-29 colorectal cancer model. Thus, we have prepared valuable model for study of tumor biology and metastatic process.

## Abbreviations

5-FU: 5-fluorouracil
ALDH: aldehyde dehydrogenase
ATP: adenosine triphosphate
CRC: colorectal carcinoma
CSC: cancer stem cells
EGFP: enhanced green fluorescent protein
SCID/bg: severe combined immunodeficiency/beige

## References

[1] Holohan C, Van Schaeybroeck S, Longley DB, Johnston PG (2013). Cancer drug resistance: an evolving paradigm. Nat Rev Cancer.

[2] Kozovska Z, Gabrisova V, Kucerova L (2014). Colon cancer: cancer stem cells markers, drug resistance and treatment. Biomed Pharmacother.

[3] Bose D, Zimmerman LJ, Pierobon M, Petricoin E, Tozzi F, Parikh A, Fan F, Dallas N, Xia L, Gaur P (2011). Chemoresistant colorectal cancer cells and cancer stem cells mediate growth and survival of bystander cells. Brit J Cancer.

[4] Romano G, Santi L, Bianco MR, Giuffre MR, Pettinato M, Bugarin C, Garanzini C, Savarese L, Leoni S, Cerrito MG (2016). The TGF-beta pathway is activated by 5-fluorouracil treatment in drug resistant colorectal carcinoma cells. Oncotarget.

[5] Huang WS, Hsieh MC, Huang CY, Kuo YH, Tung SY, Shen CH, Hsieh YY, Teng CC, Lee KF, Chen TC (2016). The association of CXC receptor 4 mediated signaling pathway with Oxaliplatin-resistant human colorectal Cancer cells. PLoS One.

[6] Yang M, Liu P, Huang P (2016). Cancer stem cells, metabolism, and therapeutic significance. Tumour Biol.

[7] Vidal SJ, Rodriguez-Bravo V, Galsky M, Cordon-Cardo C, Domingo-Domenech J (2014). Targeting cancer stem cells to suppress acquired chemotherapy resistance. Oncogene.

[8] Medema JP (2013). Cancer stem cells: the challenges ahead. Nat Cell Bioly.

[9] Chen K, Huang YH, Chen JL (2013). Understanding and targeting cancer stem cells: therapeutic implications and challenges. Acta Pharmacol Sin.

[10] Agliano A, Calvo A, Box C (2017). The challenge of targeting cancer stem cells to halt metastasis. Semin Cancer Biol.

[11] Kemper K, Grandela C, Medema JP (2010). Molecular identification and targeting of colorectal cancer stem cells. Oncotarget.

[12] Todaro M, Gaggianesi M, Catalano V, Benfante A, Iovino F, Biffoni M, Apuzzo T, Sperduti I, Volpe S, Cocorullo G (2014). CD44v6 is a marker of constitutive and reprogrammed cancer stem cells driving colon cancer metastasis. Cell Stem Cell.

[13] Pang R, Law WL, Chu AC, Poon JT, Lam CS, Chow AK, Ng L, Cheung LW, Lan XR, Lan HY (2010). A subpopulation of CD26+ cancer stem cells with metastatic capacity in human colorectal cancer. Cell Stem Cell.

[14] Vermeulen L, De Sousa EMF, van der Heijden M, Cameron K, de Jong JH, Borovski T, Tuynman JB, Todaro M, Merz C, Rodermond H (2010). Wnt activity defines colon cancer stem cells and is regulated by the microenvironment. Nat Cell Biol.

[15] Tomita H, Tanaka K, Tanaka T, Hara A (2016). Aldehyde dehydrogenase 1A1 in stem cells and cancer. Oncotarget.

[16] Chen J, Xia Q, Jiang B, Chang W, Yuan W, Ma Z, Liu Z, Shu X (2015). Prognostic value of Cancer stem cell marker ALDH1 expression in colorectal Cancer: a systematic review and meta-analysis. PLoS One.

[17] Duan JJ, Cai J, Guo YF, Bian XW, Yu SC (2016). ALDH1A3, a metabolic target for cancer diagnosis and therapy. Int J Cancer.

[18] Marcato P, Dean CA, Pan D, Araslanova R, Gillis M, Joshi M, Helyer L, Pan L, Leidal A, Gujar S (2011). Aldehyde dehydrogenase activity of breast cancer stem cells is primarily due to isoform ALDH1A3 and its expression is predictive of metastasis. Stem Cells.

[19] Qiu Y, Pu T, Li L, Cheng F, Lu C, Sun L, Teng X, Ye F, Bu H (2014). The expression of aldehyde dehydrogenase family in breast cancer. J Breast Canc.

[20] Zhang W, Liu Y, Hu H, Huang H, Bao Z, Yang P, Wang Y, You G, Yan W, Jiang T (2015). ALDH1A3: a marker of mesenchymal phenotype in gliomas associated with cell invasion. PLoS One.

[21] Cheng P, Wang J, Waghmare I, Sartini S, Coviello V, Zhang Z, Kim SH, Mohyeldin A, Pavlyukov MS, Minata M (2016). FOXD1-ALDH1A3 signaling is a determinant for the self-renewal and Tumorigenicity of mesenchymal glioma stem cells. Cancer Res.

[22] Flahaut M, Jauquier N, Chevalier N, Nardou K, Balmas Bourloud K, Joseph JM, Barras D, Widmann C, Gross N, Renella R (2016). Aldehyde dehydrogenase activity plays a key role in the aggressive phenotype of neuroblastoma. BMC Cancer.

[23] Ertem FU, Zhang W, Chang K, Mohaiza Dashwood W, Rajendran P, Sun D, Abudayyeh A, Vilar E, Abdelrahim M, Dashwood RH (2017). Oncogenic targets Mmp7, S100a9, Nppb and Aldh1a3 from transcriptome profiling of FAP and Pirc adenomas are downregulated in response to tumor suppression by Clotam. Int J Cancer.

[24] Matuskova M, Baranovicova L, Kozovska Z, Durinikova E, Pastorakova A, Hunakova L, Waczulikova I, Nencka R, Kucerova L (2012). Intrinsic properties of tumour cells have a key impact on the bystander effect mediated by genetically engineered mesenchymal stromal cells. J Gene Med.

[25] Dallas NA, Xia L, Fan F, Gray MJ, Gaur P, van Buren G 2nd, Samuel S, Kim MP, Lim SJ, Ellis LM (2009). Chemoresistant colorectal cancer cells, the cancer stem cell phenotype, and increased sensitivity to insulin-like growth factor-I receptor inhibition. Cancer Res.

[26] Kucerova L, Skolekova S, Demkova L, Bohovic R, Matuskova M (2014). Long-term efficiency of mesenchymal stromal cell-mediated CD-MSC/5FC therapy in human melanoma xenograft model. Gene Ther.

[27] Matuskova M, Kozovska Z, Toro L, Durinikova E, Tyciakova S, Cierna Z, Bohovic R, Kucerova L (2015). Combined enzyme/prodrug treatment by genetically engineered AT-MSC exerts synergy and inhibits growth of MDA-MB-231 induced lung metastases. J Exp Clin Cancer Res.

[28] Mu Y, Zang G, Engstrom U, Busch C, Landstrom M (2015). TGFbeta-induced phosphorylation of Par6 promotes migration and invasion in prostate cancer cells. Brit J Cancer.

[29] Xu P, Zhao M, Liu Z, Liu Y, Chen Y, Luo R, Fang W (2015). Elevated nuclear CCND1 expression confers an unfavorable prognosis for early stage lung adenocarcinoma patients. Int J Clin Exp Pathol.

[30] McCarroll JA, Gan PP, Erlich RB, Liu M, Dwarte T, Sagnella SS, Akerfeldt MC, Yang L, Parker AL, Chang MH (2015). TUBB3/betaIII-tubulin acts through the PTEN/AKT signaling axis to promote tumorigenesis and anoikis resistance in non-small cell lung cancer. Cancer Res.

[31] Mo X, Cao Q, Liang H, Liu J, Li H, Liu F (2016). MicroRNA-610 suppresses the proliferation of human glioblastoma cells by repressing CCND2 and AKT3. Mol Med Rep.

[32] Kanwar SS, Yu Y, Nautiyal J, Patel BB, Majumdar AP (2010). The Wnt/beta-catenin pathway regulates growth and maintenance of colonospheres. Mol Cancer.

[33] Zhao JH, Luo Y, Jiang YG, He DL, Wu CT (2011). Knockdown of beta-catenin through shRNA cause a reversal of EMT and metastatic phenotypes induced by HIF-1alpha. Cancer Investig.

[34] Redmer T, Welte Y, Behrens D, Fichtner I, Przybilla D, Wruck W, Yaspo ML, Lehrach H, Schafer R, Regenbrecht CR (2014). The nerve growth factor receptor CD271 is crucial to maintain tumorigenicity and stem-like properties of melanoma cells. PLoS One.

[35] Grunt TW, Hebar A, Laffer S, Wagner R, Peter B, Herrmann H, Graf A, Bilban M, Posch M, Hoermann G (2015). Prominin-1 (CD133, AC133) and dipeptidyl-peptidase IV (CD26) are indicators of infinitive growth in colon cancer cells. Am J Cancer Res.

[36] Dalerba P, Dylla SJ, Park IK, Liu R, Wang X, Cho RW, Hoey T, Gurney A, Huang EH, Simeone DM (2007). Phenotypic characterization of human colorectal cancer stem cells. Proc Natl Acad Sci U S A.

[37] O'Brien CA, Pollett A, Gallinger S, Dick JE (2007). A human colon cancer cell capable of initiating tumour growth in immunodeficient mice. Nature.

[38] Lee MR, Ji SY, Mia-Jan K, Cho MY (2015). Chemoresistance of CD133(+) colon cancer may be related with increased survivin expression. Biochem Bioph Res Co.

[39] Hermann PC, Huber SL, Herrler T, Aicher A, Ellwart JW, Guba M, Bruns CJ, Heeschen C (2007). Distinct populations of cancer stem cells determine tumor growth and metastatic activity in human pancreatic cancer. Cell Sem Cell.

[40] Weichert W, Knosel T, Bellach J, Dietel M, Kristiansen G (2004). ALCAM/CD166 is overexpressed in colorectal carcinoma and correlates with shortened patient survival. J Clin Pathol.

[41] Manhas J, Bhattacharya A, Agrawal SK, Gupta B, Das P, Deo SV, Pal S, Sen S (2016). Characterization of cancer stem cells from different grades of human colorectal cancer. Tumour Biol.

[42] Kucerova L, Altanerova V, Matuskova M, Tyciakova S, Altaner C (2007). Adipose tissue-derived human mesenchymal stem cells mediated prodrug cancer gene therapy. Cancer Res.

[43] Yiming L, Yunshan G, Bo M, Yu Z, Tao W, Gengfang L, Dexian F, Shiqian C, Jianli J, Juan T (2015). CD133 overexpression correlates with clinicopathological features of gastric cancer patients and its impact on survival: a systematic review and meta-analysis. Oncotarget.

[44] Amara S, Chaar I, Khiari M, Ounissi D, Weslati M, Boughriba R, Hmida AB, Bouraoui S (2015). Stromal cell derived factor-1 and CXCR4 expression in colorectal cancer promote liver metastasis. Cancer Biomark.

[45] Gunaratne A, Thai BL, Di Guglielmo GM (2013). Atypical protein kinase C phosphorylates Par6 and facilitates transforming growth factor beta-induced epithelial-to-mesenchymal transition. Mol Cell Biol.

[46] Jiao J, Zhao X, Liang Y, Tang D, Pan C (2015). FGF1-FGFR1 axis promotes tongue squamous cell carcinoma (TSCC) metastasis through epithelial-mesenchymal transition (EMT). Biochem Biophys Res Commun.

[47] de Aguiar RB, Parise CB, Souza CR, Braggion C, Quintilio W, Moro AM, Navarro Marques FL, Buchpiguel CA, Chammas R, de Moraes JZ (2016). Blocking FGF2 with a new specific monoclonal antibody impairs angiogenesis and experimental metastatic melanoma, suggesting a potential role in adjuvant settings. Cancer Lett.

[48] Sun W, Gui L, Zuo X, Zhang L, Zhou D, Duan X, Ren W, Xu G (2016). Human epithelial-type ovarian tumour marker beta-2-microglobulin is regulated by the TGF-β signaling pathway. J Transl Med.

[49] Wang HB, Yao H, Li CS, Liang LX, Zhang Y, Chen YX, Fang JY, Xu J (2017). Rise of PD-L1 expression during metastasis of colorectal cancer: implications for immunotherapy. J Dig Dis.

[50] Chen MH, Weng JJ, Cheng CT, Wu RC, Huang SC, Wu CE, Chung YH, Liu CY, Chang MH, Chiang KC (2016). ALDH1A3, the major aldehyde dehydrogenase isoform in human cholangiocarcinoma cells, affects prognosis and gemcitabine resistance in cholangiocarcinoma patients. Clin Cancer Res.

